# Pathogenesis of Eosinophilic Esophagitis: A Comprehensive Review of the Genetic and Molecular Aspects

**DOI:** 10.3390/ijms21197253

**Published:** 2020-09-30

**Authors:** Seohyun Ryu, Keum Hwa Lee, Kalthoum Tizaoui, Salvatore Terrazzino, Sarah Cargnin, Maria Effenberger, Jae Il Shin, Andreas Kronbichler

**Affiliations:** 1 Yonsei University College of Medicine, Seoul 03722, Korea; nzsarah61@gmail.com; 2 Department of Pediatrics, Yonsei University College of Medicine, Seoul 03722, Korea; AZSAGM@yuhs.ac; 3 Laboratory Microorganismes and Active Biomolecules, Sciences Faculty of Tunis, University Tunis El Manar, 1068 Tunis, Tunisia; kalttizaoui@gmail.com; 4 Department of Pharmaceutical Sciences and Interdepartmental Research Center of Pharmacogenetics and Pharmacogenomics (CRIFF), University of Piemonte Orientale, 28100 Novara, Italy; salvatore.terrazzino@uniupo.it (S.T.); sarah.cargnin@uniupo.it (S.C.); 5 Department of Internal Medicine I, Gastroenterology, Hepatology, Endocrinology and Metabolism, Medical University of Innsbruck, 6020 Innsbruck, Austria; maria.effenberger@tirol-kliniken.at; 6 Department of Internal Medicine IV (Nephrology and Hypertension), Medical University Innsbruck, 6020 Innsbruck, Austria; Andreas.Kronbichler@i-med.ac.at

**Keywords:** genetic susceptibility, pathophysiology, polymorphism

## Abstract

Eosinophilic esophagitis (EoE) is a relatively new condition described as an allergic-mediated disease of the esophagus. Clinically, it is characterized by dysphagia, food impaction, and reflux-like symptoms. Multiple genome-wide association studies (GWAS) have been conducted to identify genetic loci associated with EoE. The integration of numerous studies investigating the genetic polymorphisms in EoE and the Mendelian diseases associated with EoE are discussed to provide insights into the genetic risk of EoE, notably focusing on *CCL26* and *CAPN14*. We focus on the genetic loci investigated thus far, and their classification according to whether the function near the loci is known. The pathophysiology of EoE is described by separately presenting the known function of each cell and molecule, with the major contributors being eosinophils, Th2 cells, thymic stromal lymphopoietin (TSLP), transforming growth factor (TGF)-β1, and interleukin (IL)-13. This review aims to provide detailed descriptions of the genetics and the comprehensive pathophysiology of EoE.

## 1. Introduction

Eosinophilic esophagitis (EoE) is a relatively new condition first described in 1978; its pathology and phenotype were defined by Atwood et al. in 1993 and Straumann et al. in 1994 [1,2,3,4]. EoE is an allergy-mediated disease of the esophagus that is characterized by significant esophageal eosinophilia and esophageal dysfunction, such as dysphagia and food impaction [1,5,6,7]. Its diagnostic criteria are difficult to define because its symptoms are unspecific and mimic those observed in gastroesophageal reflux disease (GERD). Signs of damage to the esophageal barrier, such as tissue erosions and white exudates, are found in patients with EoE [8]. The disease occurs both in pediatric and adult populations, and is especially common in atopic males [9]. The overall prevalence of EoE seems to be increasing progressively [10]. This phenomenon is a consequence of better recognition patterns that are more likely to contribute to the increase in the incidence and prevalence of this condition [9,11]. Several environmental factors have been reported to contribute to the development of EoE. Twin studies have shown that the prevalence of EoE is more common in cold and dry climates, along with other allergic and autoimmune diseases [9,12,13]. Early life experiences such as premature delivery and antibiotic and acid-suppressant use have been identified to be associated with EoE [14,15,16].

Multiple genome-wide association studies (GWAS) have been conducted to identify genetic loci associated with EoE. Overexpression of several critical genes, including thymic stromal lymphopoietin (*TSLP*) and calpain 14 (*CAPN-14*), was found to disrupt the esophageal barrier and enhance immune-mediated inflammation [8,17,18]. We focus on the genetic loci investigated so far, and their classifications, depending on whether the function of the genes near the loci is known. Furthermore, the roles of inflammatory cells and various molecules, particularly TSLP, transforming growth factor (TGF)-β1, and interleukin (IL)-13, in EoE pathophysiology are summarized. This review aims to provide detailed and comprehensive information on both the genetics and pathophysiology of EoE.

## 2. Definition and Diagnosis of EoE

EoE is defined as a chronic, immune/antigen-mediated esophageal disease characterized clinically by symptoms related to esophageal dysfunction and histologically by eosinophil-predominant inflammation [19,20].

For the last decade, diagnosis of EoE required an esophageal biopsy with 15 or more eosinophils present in one high-power field even with an eight week or longer treatment with maximal dose proton pump inhibitor (PPI). The recently updated consensus criteria in 2017 removed the PPI trial for the diagnosis of EoE. Although ~50% of EoE patients show PPI-responsive esophageal eosinophilia (PPI-REE), it is agreed that as PPI is a treatment for EoE, there is no need to diagnose the disease primarily based on response to treatment [19]. As such, the updated diagnostics criteria for EoE requires symptoms of esophageal dysfunction, ≥15 eosinophils per high-power field (~60 eos/mm^2^) on an esophageal biopsy, and an assessment of non-EoE disorders that cause or potentially contribute to esophageal eosinophilia (Box 1) [6,19,21].

Box 1 **Secondary causes of eosinophilic esophagitis (EoE).**

Eosinophilic gastrointestinal diseases

Gastroesophageal reflux disease (GERD)

Celiac disease

Crohn’s disease

Infection

Hypereosinophilic syndrome

Achalasia

Drug hypersensivity

Vasculitis

Pemphigus

Connective tissue disease

Graft vs. host disease

## 3. Genetics

The EoE transcriptome is a distributed section throughout the human genome, which shows a conserved expression in the esophagus of patients with EoE [22,23]. The EoE transcriptome has 574 genes expressed differently in tissues of children [2,24]. Studying genetic variants in EoE transcriptome provides a deep understanding of the mechanisms of EoE, and there are still many genes remaining with an unknown role in pathophysiology. The most highly expressed gene in the EoE transcriptome is *CCL26*, the expression of which is induced by IL-13 [22,25,26]. The strongest transcriptional changes occur at *1q21*, which encodes the epidermal differentiation complex [22]. Genes included in *1q21*, such as filaggrin, show considerably minimal activity in EoE, which leads to the loss of epidermal cell differentiation and impaired barrier function [22,27,28].

### 3.1. Risk Genes

Candidate gene studies regarding EoE, GWAS, and analysis of its association with other monogenic disorders are the main methods used to study the genetics of EoE [9]. These studies aim to identify genetic loci that may contribute to EoE. Some of the genes have known functions, while others are still unknown [14]. Some genetic risk loci are found in most EoE patients, while some are rare even among these patients. In this review, we present identified, common, and rare genetic risk loci and their known functions.

#### 3.1.1. Common Risk Genes and Function

Table 1 summarizes the common risk genes identified thus far that have known functions with further information on genetic risk loci, the *p*-value in the case of the GWAS approach, and the name of another approach if used instead of GWAS (this is highlighted inside the bracket). Some of the genes not described in this section are instead described with regard to EoE-associated diseases in Section 3.2.

When allergens are exposed to the esophageal epithelium, epithelial cells and basophils produce TSLP [2,29,30]. TSLP plays an important role in promoting Th2 cell differentiation by inducing the Th2-polarizing capacity of dendritic cells [22,31]. A *TSLP* single nucleotide polymorphism (SNP) augments the Th2 response [2]. TSLP levels are significantly higher in patients with atopic disease, including EoE [22,32]. In addition, SNPs in a gene encoding a component of the TSLP receptor, *CRLF2*, are associated with increased EoE risk [33]. Activated Th2 cells produce cytokines such as IL-4, IL-5, and IL-13. IL-4 promotes the differentiation of T cells into Th2 and B cells, eventually leading to IgE secretion [2,34]. IL-5 and IL-13 induce the secretion of eotaxin-3 from epithelial cells [26,29]. The eotaxin-3 gene, which induces eosinophil recruitment to the esophagus, has the strongest transcriptome expression levels, i.e., approximately 53 times higher than in controls [2,35]. IL-13 also reduces the expression levels of genes in the epidermal differentiation complex such as filaggrin and involucrin, thus weakening the barrier function of the squamous epithelium [29,36]. At the same time, locally activated eosinophils and mast cells produce TGF-β1, which triggers fibrotic changes in the esophageal wall, which in turn is mediated by fibroblasts and periostin, thus leading to smooth muscle dysfunction [24,29]. A SNP in the promoter of TGF-β1 is responsible for esophageal dysfunction [2].

*CAPN* encodes a proteolytic enzyme specifically in the esophagus [18,22]. IL-13 induces the activity of this enzyme, and CAPN14 invokes a pathway that alters basic epithelial cell functions, including barrier integrity [18,22]. STAT6 is known to be an IL-13-activated transcription factor; it induces CAPN expression [37]. Thus, SNPs in *CAPN14* and *STAT6* are common genetic risk factors in EoE. CAPN14 has also been identified as a regulator of desmoglein 1(DSG1) [38]. DSG1 regulates esophageal epithelial barrier function and immune responses [28]. DSG1 is decreased in EoE and is associated with an impaired barrier phenotype [9,28]. FLG is associated with esophageal barrier integrity maintenance [33,36,39].

*LRRC32,* which encodes a TGF-β binding protein, and *C11orf30*, which encodes EMSY, are involved in transcriptional regulation [22]. EMSY and LRRC32 are both expressed in esophageal epithelial cells; however, the roles of these proteins in EoE are yet to be reported [38].

19q13 is another genetic risk locus, and the genes *ANKRD27*, *PDCD5*, and *RGS9BP* are near this location. The protein ANKRD27 inhibits the activity of the SNARE complex, which could have important implications for apical transport in esophageal epithelial cells and in wound healing [14,40,41]. The PDCD5 protein is known to be involved in apoptotic pathways, transcriptional regulation, DNA damage response, and cell cycle control [42]. However, *RGS9BP* encodes a product not expressed in the esophagus and immune cells [14,43,44]. Further studies are needed to identify the role of these genes, and their associated proteins, in EoE.

Several other genetic risk loci were identified by GWAS, but their functions remain obscure. The locus, tag of genetic variant, and *p* value of each gene are specified in Table 2 [14,45,46,47,48]. These genes may have yet to be discovered functions that contribute to the genetic mechanism of EoE.

#### 3.1.2. Rare Risk Genes and their Function

Studies on risk genes rarely found in EoE patients are emerging. Rochman et al. identified 39 rare variants by performing whole-exome sequencing (WES) in 33 patients; these variants have the potential to alter the biological function of EoE-associated genes [14,27]. Sherrill et al. also performed WES in 63 patients, focusing particularly on families, identifying 5 rare, damaging variants in dehydrogenase E1 and transketolase domain-containing 1 (*DHTKD1*) [14,49]. Conducting careful studies to identify novel rare genetic variants of EoE will provide insights into the complex pathophysiology of EoE and associated diseases.

### 3.2. Associated Diseases of EoE

EoE is often studied with associated Mendelian diseases. Table 3 summarizes the Mendelian diseases associated with EoE [14,22,38]. Studying these co-occurring diseases might help to identify certain genes and the corresponding pathogenic mechanisms of EoE.

Connective tissue disorders (CTDs) (e.g., Loeys-Dietz syndrome (LDS) and Ehlers-Danlos syndrome (hypermobility type)) are the most well-recognized diseases associated with EoE [9,22,50]. A diagnosis of EoE increases the risk of developing a CTD by eight-fold [34,38]. Increased production and/or signaling of TGF-β and dysregulated expression of collagen in the esophagus commonly occur in both CTD and EoE [22,38,51,52,53]. Specifically, LDS is caused by gain-of-function mutations in the TGF-β receptors, *TGFBR1*, and *TGFBR2*, whereas Ehlers-Danlos syndrome is caused by genetic mutations in collagen-encoding genes [9,38,54,55,56].

Severe atopy syndrome associated with metabolic wasting (SAM syndrome) also co-occurs with EoE [22]. Downregulation of *DSG1* in the esophageal epithelia is reported in both SAM syndrome and EoE [2,8,38]. DSG1 is a major constituent of desmosomes; thus, downregulation of DSG1 leads to the impaired barrier phenotype [9,28].

EoE is enriched in patients with Netherton’s syndrome, which is caused by autosomal dominant loss-of-function mutations in the protease inhibitor *SPINK5* [22,38,57,58]. Without *SPINK5*, which is a regulator of the epidermal proteases kallikrein-related peptidase KLK5 and KLK7, the skin is disrupted substantially [38,59]. The association between EoE and Netherton’s syndrome shows that barrier impairment has a central role in both diseases [38].

PTEN hamartoma tumor syndrome (PHTS) is associated with EoE [9,60]. PHTS carries a >200-fold increased risk for eosinophil-associated gastrointestinal disorders, including EoE [9,60]. PHTS is caused by mutations in the tumor suppressor *PTEN*, which is a critical regulator of the phosphatidylinositol-4,5-biphosphate 3-kinase (PI3K) pathway [38,61]. Moreover, eosinophils are capable of expressing PTEN [38].

Autosomal-dominant hyper-IgE syndrome, caused by deleterious mutations in *STAT3*, is associated with EoE [4]. Deleted function of STAT3 leads to dysregulated response to IL-6, which causes deficit in T-helper 17 cells, central T cell memory, and memory B cells [13,38]. STAT3 is activated in eosinophils following IL-5 signaling, but the role of eosinophils in hyper-IgE is yet to be discovered [13,38].

Autosomal-recessive hyper-IgE syndrome, caused by loss-of-function mutations in *DOCK8*, is also associated with EoE [9,62]. DOCK8, which is expressed on human eosinophils, functions in T-cell homeostasis, and in a durable secondary antibody response [38,63]. It maintains the morphological shape and nuclear integrity of T and NK cells during chemotaxis, through CDC42 and p21-activated kinase (PAK) [38,64].

In addition, ERBB2-interacting protein (ERBIN) deficiency is related to EoE [22,65]. *ERBIN* downregulates TGF-β signaling [22,65]. EoE is also known to be associated with esophageal granular cell tumors, but it is uncertain whether this is a disease association or consequence of EoE [22,66].

## 4. Pathophysiology

**Figure 1:**
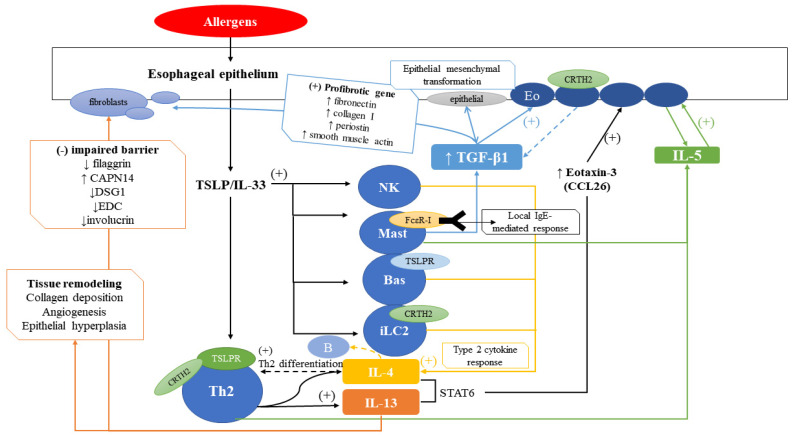
Overview of EoE pathophysiology. Allergens stimulate the esophageal epithelium, inducing TSLP/IL-33, leading to stimulation of Th2 cells, NK cells, mast cells, basophils, and iLC2. Main receptors on each cell are indicated. NK cells, mast cells, basophils, iLC2, and Th2 cells induce IL-4 which induce Th2 differentiation. IL-4 and IL-13 induced by Th2 cells induce eotaxin-3 (CCL26), which stimulates eosinophils to secrete IL-5. IL-5, secreted by Th2 cells and mast cells, also stimulate eosinophils. Mast cells also induce TGF-β1 which stimulate eosinophils and fibroblasts, as outlined in the blue box. IL-13 induces impaired barrier function and tissue remodeling, as outlined in the orange box.

EoE is caused by an allergic inflammation reaction in patients that have genetic and environmental risks of EoE, and it relies on both the innate and adaptive immune pathways. Thus, the underlying pathophysiology of EoE is complex and diverse pathways are involved, with many immune cells or cytokines contributing to this disease. Figure 1 shows a compact overview of the EoE pathophysiology. Recently, approximately 50% of EoE patients were found to fall into the category of PPI-REE; therefore, the pathophysiological characteristics to distinguish PPI-REE from EoE are important future research fields [19,67].

### 4.1. Role of Inflammatory Cells

#### 4.1.1. Eosinophils

Eosinophils are recruited from the blood with local chemotaxis and they seem to be integral to EoE disease pathogenesis [2,68,69,70,71]. Eosinophils release eosinophilic peroxidase (EPO), eosinophil cationic protein (ECP), and major binding protein (MBP), which directly causes tissue damage and esophageal dysmotility [2,9,72]. ECP damages cellular membrane barriers, and MBP increases smooth muscle reactivity by causing the dysfunction of vagal muscarinic M2 receptors, while also provoking mast cell and basophil degranulation [2,9]. Eosinophils also serve as antigen-presenting cells (APCs) with MHC-II presentation as well as co-stimulatory molecules (CD40, CD28, CD86, and CD27) [1,9,73]. Eosinophils secrete a variety of cytokines (IL-2, IL-4, IL-6, IL-10, IL-12) which together can activate T cells [9,73]. Eosinophils also produce IL-1, IL-3, IL-4, IL-5, IL-13, TGF-β, eotaxin-3, RANTES, macrophage inflammatory protein 1 (MIP-1), tumor necrosis factor (TNF)-α, granulocyte-macrophage colony-stimulating factor (GM-CSF), platelet-activating factor (PAF), and leukotriene C4 (LTC4) [1,9].

The importance of eosinophils in EoE has been studied in both mouse and human models. Mice genetically engineered to lack eosinophils and mice in which eosinophils are selectively targeted via antibody treatment showed a decrease in the symptoms in many, but not all features [9,69,70,74]. This implies that an EoE-like disease is not completely dependent on eosinophils. In vivo, experimental adoptive transfer of antigen-pulsed eosinophils produced antigen-specific T cell responses, which proves T cell activation by eosinophils [9].

#### 4.1.2. T Cells

In EoE, the numbers of CD3+, CD4+, and CD8+ T cells, as well as the CD8+/CD4+ T cell ratio, increase in the esophageal mucosa [1,9,75,76]. As observed in case of many other allergic reactions, Th2 cells are mainly involved in the inflammatory response [9]. Higher abundances of pathogenic effector Th2 cells (peTh2 cells) were detected in patients with EoE; which were chemoattractant receptor-homologous molecule-positive (CRTH2+), hematopoietic prostaglandin D synthase-positive (HPSD+), and CD161 high CD4+ T cells [22,77,78]. CRTH2 was present on peTH2 cells, eosinophils, and basophils and in response to prostaglandin D2 changed the chemotaxis of these cells [22]. Th2 cells produce type 2 cytokines such as IL-4, IL-5, and IL-13, which play a key role in the pathogenesis of EoE. In murine models, recombination activation gene 1 (RAG1) knockout mice were completely protected from experimentally derived EoE, whereas CD4 knockout mice were partially protected and CD8 and B cell knockout mice were not protected [1,9,79].

Regulatory T cells were increased in esophageal biopsies, but not in the percentage of total T cells; thus, their significance in EoE is unclear [1].

#### 4.1.3. Mast Cells

The esophageal mast cell content increased significantly, and mast cell degranulation was detected in nearly all patients with EoE [1,33,80,81]. Genes specific to mast cells, such as those that encode carboxypeptidase 3A (CPA3), FcεR-I, and tryptase (TPSAB1), were highly expressed in the EoE transcriptome [1,9]. IgE was bound by the FcεR-I receptor in the membrane of mast cells, and IgE may have contributed to a local IgE-mediated immediate hypersensitivity response in the esophagus [9,80]. Mast cells secrete diverse products such as cytokines, proteases, and bioactive compounds, and many of these products lead to esophageal remodeling and dysmotility [9,33].

In murine models, mast cell numbers and eosinophil numbers increased as EoE symptoms increased [1,81]. Mice with depleted IgE and mast cells still showed EoE-like symptoms, but they showed reduced muscle cell hyperplasia and hypertrophy [9,81,82]. This implied that although mast cells were not required in experimentally derived EoE, they were required to increase the thickness of the muscularis mucosa [33]. Further studies in murine models have found that both IL-5 and IL-9 transgenic mice had increased numbers of mast cells, suggesting that both IL-5 and IL-9 promote mast cell activation and maturation [1,83]. A clinical trial using IL-5 antibodies showed that mast cell numbers were correlated with EoE symptom severity, while eosinophil numbers were not [9,84].

#### 4.1.4. Basophils

Basophils are known to play a key role in allergic responses. Increased esophageal basophilia was observed in EoE, and these basophils showed increased ST2 expression [1,85,86]. Basophils have been reported to secrete various type 2 cytokines and act as antigen presenting cells (APCs) to induce Th2 cells [33,87]. In particular, basophils expressed the receptor of TSLP (TSLPR) [2,88]. Basophil-deficiency led to the prevention of EoE; furthermore, TSLP and basophils are required to maintain EoE after disease establishment [89].

By observing EoE biopsies, Siracusa et al. showed that TSLP affected basophil hematopoiesis [86]. In a murine model of EoE, Noti et al. suggested that basophils were critical to eosinophil recruitment via TSLP [1,90]. In addition, in a TSLP-dependent, experimentally derived EoE model, an increased number of basophils were recruited in an ST2-dependent manner, implying that IL-33 may induce basophil recruitment [33,91].

#### 4.1.5. Dendritic Cells

Dendritic cells normally reside in the esophageal epithelium and an increased number of these cells were found in patients with EoE [33,75,76]. There was evidence that dendritic cells may present antigens in EoE [9,79,92,93]. Langerhans cells, a type of dendritic cells found in the esophagus, expressed FcεRI, which was correlated with the Th2 response level [9,94]. These Th2 responses were induced by allergens and/or environmental adjuvants, likely via communication between resident stromal and dendritic cells [9,95,96].

#### 4.1.6. Innate Lymphoid Cells

Innate lymphoid cells (ILCs) are resident immune cells in tissues that may serve as sources of type 2 cytokines [9,33,97,98,99]. Group 2 ILCs (ILC2s) expressed CRTH2 and secreted large quantities of type 2 cytokines in response to IL-25, IL-33, and TSLP [9].

In murine models, ILCs were important in infection and inflammation responses, as well as in tissue repair of EoE [9,99]. Doherty et al. reported that ILC2s were present in EoE biopsies, with an increased level in active EoE, and this correlated with the number of eosinophils found in biopsies [9,99].

#### 4.1.7. Invariant Natural Killer T Cells (iNKT Cells)

Invariant natural killer T cells (iNKT cells) recognize lipid and glycolipid antigens that are presented by CD1d molecules, and they have the capacity to produce type 2 cytokines [9,33,100,101,102]. Mucosal iNKT tolerance to environmental antigens can mediate allergic sensitization and tissue inflammation in the absence of tolerance [9,103].

In murine models, CD1d-deficient mice were protected from experimental EoE, and activation of iNKT was sufficient to induce EoE. Furthermore, iNKT neutralized mice were also protected from experimental EoE [9,104,105]. Recently, a possible role of iNKT cells in protecting from EoE-specific pathologies was shown in RAG1-deficient mice [9]. Fewer iNKT cells were found in the peripheral blood of patients, while increased numbers of iNKT were found in the esophagus [33,105,106]. iNKTs from patients expanded more readily and produced more IL-13 in response to stimulation [9,106].

#### 4.1.8. B Cells

Mouse models of EoE showed that B cell-deficient mice still developed EoE, suggesting that EoE does not rely on B cells [1,79].

### 4.2. Role of Various Molecules

#### 4.2.1. TSLP

TSLP and its receptor TSLPR are implicated in various EoE pathways. TSLP expression was increased in esophageal tissues in patients with EoE [89]. TSLP mainly induced a type 2 immune response [89]. TSLPR-deficient mice were protected from experimentally derived EoE [89,90].

#### 4.2.2. TGF-β1

TGF-β1, produced by mast cells, eosinophils, and esophageal epithelial cells, is a key cytokine for epithelial fibrosis and epithelial cell transformation [9]. Elevated expression of TGF-β1 was found in the esophageal biopsy samples of patients with active EoE when compared to that in samples from control patients or patients with GERD [9,70,107,108,109].

TGF-β1 can have profibrotic effects on esophageal fibroblasts [89] and TGF-β1 can directly induce the expression of profibrotic genes such as fibronectin, collagen I, periostin, and smooth muscle actin in EoE fibroblasts [89]. Studies on murine models support this function; the TGF-β1 pathway mediator SMAD2/3 was important in esophageal fibrosis, and SMAD3-deficient mice were partially protected from EoE-associated fibrosis [68,110]. TGF-β1 also affects associated cellular functions. TGF-β1 can alter the contraction of collagen gels in esophageal smooth muscle cells [52,53,68]. This function has recently been found to rely on the expression of phospholamban (PLN), a protein that regulates calcium flux [52,68]. EoE esophageal smooth muscles expressed PLN, while the absence of PLN was found in controls [52,68]. Inhibition of PLN expression and signaling through TGF-β receptor I both decreased esophageal smooth muscle contraction in response to TGF-β1 [52,68].

Studies of human epithelial cells showed that TGF-β1 induced epithelial mesenchymal transformation, with increased vimentin expression [68,111]. The degree of epithelial mesenchymal transformation correlated positively with both TGF-β1 expression and eosinophil numbers [53,68,107].

#### 4.2.3. IL-4

IL-4 is secreted by Th2 cells, NK cells, and TSLP-dependent basophils [2,34]. IL-4, but not IL-13, induced Th2 cell differentiation, differentiation of naive T cells into Th2 and active B cells class switching to produce IgE [9].

#### 4.2.4. IL-5

IL-5 is secreted by Th2 cells, mast cells, and eosinophils [9,112]. IL-5 promotes eosinophil proliferation, survival, activation, and chemotaxis [1]. IL-5 and its receptor were expressed in esophageal tissue in EoE, and it increased the IL-5 mRNA and protein expression levels, which were observed in the esophagus of EoE patients [89,93]. Previous studies of anti-IL-5 therapy in humans were not effective in improving the symptoms of EoE, although protective effects against esophageal eosinophilia were observed [9,109].

#### 4.2.5. IL-13

IL-13, a key cytokine and the most studied cytokine in EoE pathogenesis, is secreted by Th2 cells and activates eosinophils [9,93,113,114]. Esophageal IL-13 overexpression by Th2 cells induces CCL26, eotaxin-3, and periostin expression, eosinophilic recruitment by upregulation of an eosinophil chemokine, and CAPN14 expression, which was responsible for STAT6 and IL-33 production [2,34]. IL-13 downregulates the expression of DSG-1, filaggrin, EDC, and involucrin, which are proteins important in epithelial integrity and barrier function [9,28,36]. In addition, independent of eosinophilia, IL-13 induces tissue remodeling by promoting collagen deposition, angiogenesis, and epithelial hyperplasia [9,71].

In murine models, IL-13 induced EoE and tissue remodeling whereas IL-13-deficient mice showed improvements in esophageal symptoms [9,71,115]. In human trials, anti-IL-13 reduces tissue eosinophilia [1,116,117].

#### 4.2.6. IL-15

An increased amount of IL-15 is observed in patients with EoE and in murine models [9]. IL-15 contributes to CD4+ T and iNKT cell growth, including the synthesis of IL-5 and IL-13 in EoE [9,25,36,70,118].

#### 4.2.7. Eotaxin-3 (CCL26)

Eotaxin-3, mainly produced by esophageal epithelial cells through the IL-13 signaling pathway, and is implicated in eosinophil trafficking to the esophagus in patients with EoE [9,26]. Eotaxin-3 is the most abundant EoE chemokine regardless of the age, sex, and atopic status of the patient [1,26,119]. Microarray analysis showed that eotaxin-3 has the largest fold change in mRNA expression level between patients with EoE and controls [89].

In murine models, mice lacking the eotaxin receptor CCR3 were protected from developing experimental EoE [9,26].

#### 4.2.8. IgE and IgG4

IgE is a cytokine that contributes to many atopic pathways; however, growing evidence suggests that IgE has no direct role in EoE [89]. B cell-deficient mice still developed EoE [1,79]. IgE was not elevated in all patients with EoE and omalizumab, an anti-IgE monoclonal antibody, was ineffective in the treatment of EoE [89,120].

Increased levels of IgG4 were observed in EoE esophageal tissues [89,120,121]. However, the specific contribution of IgG4 in EoE is yet to be discovered [89].

#### 4.2.9. Prostaglandins

Prostaglandins affect the eosinophil pathway in the esophagus [9,122]. Prostaglandin inhibitors showed protective effects against EoE-like inflammation [9,123]. The chemoattractant receptor (CRTH2) expressed on Th2 cells is the receptor for prostaglandin D2 (PGD2) and mediated the chemotaxis of Th2 cells, eosinophils, and basophils [9,123].

#### 4.2.10. Additional Cytokines

Several studies have identified additional cytokines in the EoE pathway. Collison et al. found that TNF-related, apoptosis-inducing ligand (TRAIL) controlled MID1 and TSLP expression, inflammation, fibrosis, smooth muscle hypertrophy, and expression of cytokines in experimentally derived EoE. These included cytokines such as TSLP, CCL11, CCL20, CCL24, IL-5, IL-13, IL-25, and TGF-β [9,124]. De Souza et al. identified that macrophage migration inhibitory factor (MIF) induced eosinophil infiltration and remodeling in EoE in a murine model [9,125]. Dutt et al. identified that allergen-induced IL-18 promoted IL-5-and iNKT-dependent EoE pathology [9,126].

## 5. Summary

Eosinophilic esophagitis (EoE) is a relatively new allergy-mediated condition. In this comprehensive review, we focused on providing a detailed description of both common and rare genetic risk loci of EoE. The function of various common and rare genetic loci remains unknown (Table 2, Section 3.1.2.), and further genetic studies should focus on revealing these roles. The pathophysiology of EoE is complex, with a network of various cells and molecules contributing to it, especially eosinophils, Th2 cells, TSLP, TGF-β1, and IL-13. Molecules that have not yet been identified may contribute to the mechanism of EoE. Moreover, although patients with PPI-REE account for about 50% of patients with EoE, the distinguishing genetics and pathophysiology have not yet been identified and should be investigated further.

## Figures and Tables

**Table 1:** Common risk genes with known functions.

Genetic Risk Loci	Gene at and Near Risk Variants	*p*-Value in Case of GWAS Approach (or the Approach Used)	Known Function/Possible Pathogenic Mechanisms
2p23.1	*CAPN14*	5 × 10^−10^	Encodes a proteolytic enzyme specific to the esophagus that is induced by IL-13 and involved in epithelial homeostasis and repair
	*CCL26* (/*eotaxin-3*)	(candidate gene)	A potent eosinophil chemoattractant and activating factor induced by IL-13
	*CRLF2*	(candidate gene)	TSLP receptor
	*FLG*	(candidate gene)	Esophageal barrier function
	*IL-5/IL-13*	(PheWAS)	Th2 signaling
11q13.5	*LRRC32* *C11orf30 (EMSY)* *CAPN5*	4 × 10^−11^	LRRC32 is a TGF-β binding protein. Possibly TFG-β signaling/epithelial protease function/barrier function EMSY is involved in transcriptional regulation
12q13	*STAT6*	2 × 10^−6^	IL-13 responsive transcription factor, Th2 development
	*TGFβ1*	(candidate gene)	Th2 skewing and fibrosis
5q22.1	*TSLP* *WDR36*	3 × 10^−9^	Potent Th2 skewing Induces Th2 cell development and activates eosinophils and basophils
19q13.11	*ANKRD27* *PDCD5* *RGS9BP*	2 × 10^−9^	ANKRD27 inhibits the SNARE complex PDCD5 is involved in apoptotic pathways RGS9BP is not expressed in the esophagus or by immune cells
18q12.1	*DSG1* *DCC*	(mapping/sequencing/phenotype association) 7 × 10^−6^	Regulates esophageal epithelial barrier function
	*TGFβR1*/*TGFβR2*/*PBN*	(phenotype association)	Th2 skewing and fibrosis
	*PTEN*	(phenotype association)	Regulation of eosinophil response
	*STAT3*	(phenotype association)	Engagement in signal pathway of growth factors, hormones and multiple cytokines
	*SPINK5*	(phenotype association)	Esophageal barrier function
	*DOCK8*	(phenotype association)	Potent role in T-cell homeostasis

GWAS, genome-wide association studies. CAPN14, calpain-14. IL, interleukin. CCL26, chemokine ligand 26. CRLF2, cytokine receptor-like factor 2. TSLP, thymic stromal lymphopoietin. FLG, filaggrin. PheWAS, phenome-wide association studies. LRRC32, leucine-rich repeat containing 32. STAT, signal transducer and activator of transcription. TGFβ, transforming growth factor beta. WDR, WD repeat domain. ANKRD, ankyrin repeat domain. PDCD, programmed cell death. RGS9BP, regulator of G protein signaling 9 binding protein. DSG, desmoglein. DCC, deleted in colorectal cancer. FBN, fibrillin. PTEN, phosphatase and tensin homolog. STAT, signal transducer and activator of transcription. SPINK5, serine protease inhibitor Kazal-type 5. DOCK8, dedicator of cytokinesis 8.

**Table 2:** Common risk genes with unknown functions.

Genetic Risk Locus	Tag Genetic Variant	Genes at and Near Risk Variants	*p*-Value
1p13.3	rs2000260	*SLC25A24*	7 × 10^−7^
1p36.13	rs28530674	*KIF17*	3 × 10^−7^
	rs2296225		1 × 10^−7^
1p32.2	rs11206830	*AC119674.2*	8 × 10^−8^
	rs77569859		3 × 10^−10^
3q26.32	rs6799767		4 × 10^−7^
4q21.1	rs13106227	*SHROOM3*	4 × 10^−6^
	rs1986734		1 × 10^−6^
	rs3806933		2 × 10^−8^
	rs252716		4 × 10^−14^
5q23.1	rs2055376	*SEMA6A*	7 × 10^−8^
5q14.2	rs1032757		2 × 10^−6^
6p11.2	rs9500256	*AL445250.1*	5 × 10^−6^
8p23.1	rs2898261	*XKR6*	5 × 10^−8^
8q24.12	rs11989782	*SNTB1*	7 × 10^−6^
8q22.2	rs13278732	*ERICH5*	6 × 10^−6^
10p12.31	rs11819199	*MIR4675*	3 × 10^−7^
10q23.1	rs2224865	*MARK2P15-LINC02650*	9 × 10^−6^
	rs2155219		4 × 10^−7^
	rs77301713		1 × 10^−7^
11q14.2	rs118086209	*CCDC81*	2 × 10^−7^
11q21	rs1939875	*NR*	3 × 10^−6^
14q12	rs8008716	*NOVA1*	7 × 10^−8^
15q13.3	rs8041227	*LOC283710*, *KLF13*	6 × 10^−10^
16p13	rs12924112	*CLEC16A*	2 × 10^−9^
16q24.1	rs371915	*MEAK7*	2 × 10^−8^
17q24.3	rs6501384	*CALM2P1-AC011990.1*	6 × 10^−6^
17q25.3	rs3744790	*TIMP2*, *CEP295NL*	8 × 10^−7^
	rs9956738		4 × 10^−7^
21q22.3	rs17004598	*HSF2BP*	1 × 10^−7^
22q11.21	rs2075277	*P2RX6*	9 × 10^−7^

SLC25A24, solute carrier family 25 member 24. KIF17, kinesin-like protein 17. SHROOM3, shroom family member 3. SEMA6A, semaphorin 6A. XKR6, XK related 6. SNTB1, syntrophin beta 1. ERICH5, glutamate-rich protein 5. MIR4675, microRNA 4675. MARK2P15, microtubule affinity regulating kinase 2 pseudogene 15. LINC02650, long intergenic non-protein-coding RNA 2650. CCDC81, coiled-coil domain-containing protein 81. NOVA1, the NOVA alternative splicing regulator 1. KLF13, kruppel-like factor 13. CLEC16A, a C-type lectin domain containing 16A. MEAK7, MTOR associated protein, Eak-7 homolog. CALM2P1, calmodulin 2 pseudogene 1. TIMP2, a tissue inhibitor of metalloproteinases 2. CEP295NL, CEP295 N-terminal. HSF2BP, a heat shock transcription factor 2 binding protein. P2RX6, purinergic receptor P2X 6.

**Table 3:** Mendelian diseases associated with EoE.

Mendelian Disease Associated with EoE	Genetic Mutation	Plausible Etiologic Mechanism
Loeys-Dietz syndrome (LDS)	Mutations in *TGFBR1* and *TGFBR2*	Enhanced TGF-β signaling
Ehlers-Danlos syndrome, hypermobility type	Unknown; other subtypes of Ehlers-Danlos syndrome are caused by mutations in collagen genes	Disrupted joint and skin development; increased activity of TGF-β due to altered binding by extracellular matrix
Severe atopy syndrome associated with metabolic wasting (SAM syndrome)	Homozygous mutations in *DSG1*	Disrupted epithelial barrier
Neherton syndrome	Loss-of-function mutations in *SPINK5*	Unrestricted protease activity of KLK5 and KLK7
PTEN hamartoma tumor syndrome (PHTS)	Mutations in *PTEN*	Inhibited regulation of the phosphatidylinositol-4,5-biphosphate 3-kinase (PI3K) signaling pathway
Autosomal dominant hyper-IgE syndrome	Deleterious mutations in *STAT3*	Dysregulated response to IL-6 and possibly IL-5
Autosomal recessive hyper-IgE syndrome	Loss-of-function mutations in *DOCK8*	Loss of T-cell homeostasis; lack of durable secondary antibody response against specific antigens
ERBIN deficiency	Loss-of-function mutation in *ERBIN*	Increased TGF-β pathway activation in T cells with increased Th2 responses

TGFBR, transforming growth factor beta receptors. TGF-β, transforming growth factor beta. DSG1, desmoglein-1. SPINK5, serine protease inhibitor Kazal-type 5. KLK5, kallikrein-related peptidase 5. PTEN, phosphatase and tensin homolog. STAT3, a single transducer and activator of transcription 3. IL, interleukin. Ig, immunoglobin. DOCK8, a dedicator of cytokinesis 8. ERBIN, Erbb2 interacting protein.
